# Intensity and timing of physical activity in relation to postmenopausal breast cancer risk: the prospective NIH-AARP Diet and Health Study

**DOI:** 10.1186/1471-2407-9-349

**Published:** 2009-10-01

**Authors:** Tricia M Peters, Steven C Moore, Gretchen L Gierach, Nicholas J Wareham, Ulf Ekelund, Albert R Hollenbeck, Arthur Schatzkin, Michael F Leitzmann

**Affiliations:** 1 Nutritional Epidemiology Branch, Division of Cancer Epidemiology and Genetics, National Cancer Institute, NIH, DHHS, 6120 Executive Blvd, Bethesda, MD, USA; 2 MRC Epidemiology Unit, Institute of Metabolic Science, Addenbrooke's Hospital, Hills Rd, Cambridge, UK; 3 Hormonal and Reproductive Epidemiology Branch, Division of Cancer Epidemiology and Genetics, National Cancer Institute, NIH, DHHS, 6120 Executive Blvd, Bethesda, MD, USA; 4 Cancer Prevention Fellowship Program, Office of Preventive Oncology, National Cancer Institute, NIH, DHHS, Bethesda, MD, USA; 5 AARP, 601 E St NW, Washington, DC, USA; 6 Department of Epidemiology and Preventive Medicine, University Medical Center Regensburg, Regensburg, Germany

## Abstract

**Background:**

Despite strong evidence of an inverse association of physical activity with postmenopausal breast cancer risk, whether a certain intensity or time of life of physical activity is most effective for lowering breast cancer risk is not known.

**Methods:**

In 118,899 postmenopausal women in the prospective NIH-AARP Diet and Health Study, we examined the relations of light and moderate-to-vigorous intensity physical activity during four periods of life ("historical": ages 15-18, 19-29, 35-39 years; "recent": past 10 years) to postmenopausal breast cancer risk. Physical activity was assessed by self-report at baseline, and 4287 incident breast cancers were identified over 6.6 years of follow-up.

**Results:**

In age-adjusted and multivariate Cox regression models, >7 hours/week of moderate-to-vigorous activity during the past 10 years was associated with 16% reduced risk of postmenopausal breast cancer (RR:0.84; 95%CI:0.76,0.93) compared with inactivity. The association remained statistically significant after adjustment for BMI (RR:0.87; 95%CI:0.78,0.96). Neither moderate-to-vigorous activity during other periods of life nor light intensity activity during any period of life was related to breast cancer risk, and associations did not vary by tumor characteristics.

**Conclusion:**

A high level of recent, but not historical, physical activity of moderate-to-vigorous intensity is associated with reduced postmenopausal breast cancer risk. More precise recall of recent physical activity than activity in the distant past is one possible explanation for our findings.

## Background

With an estimated 182,460 new cases diagnosed in the United States (U.S.) in 2008 [1], breast cancer is recognized as the most common cancer affecting U.S. women. Among the few modifiable risk factors for breast cancer, a high versus low level of physical activity has been consistently associated with 20-40% reduced risk of postmenopausal breast cancer [2-5]. In order to better characterize the physical activity-breast cancer relation, further investigation of specific parameters of physical activity such as the intensity and the time of life of physical activity that may be most effective for breast cancer prevention among postmenopausal women is needed [3,5].

Some studies suggest that physical activity of vigorous intensity is associated with a greater reduction in breast cancer risk than low intensity activity [6-9], and a recent review [5] reported a more pronounced risk reduction for vigorous (26%) than moderate (22%) physical activity. Other investigations have not found a dose-response relation according to activity intensity levels [10-18].

In addition, two recent reviews [3,5] reported stronger inverse associations with breast cancer risk for adult activity than for activity during adolescence. However, supporting evidence from prospective studies among postmenopausal women is limited to a few studies [12,19,20]. Observation of a certain time period of life during which physical activity most strongly influences postmenopausal breast cancer risk would have substantial relevance for targeting age-specific interventions to increase activity.

Intensity- and/or age-dependent variation in the physical activity-breast cancer relation could also help inform the etiology of breast cancer. Several biological mechanisms by which physical activity is suggested to act on breast tumor development have been proposed [21,22], including lowering levels of endogenous sex hormones [23], modulating insulin and insulin-like growth factors, enhancing immunity, and reducing chronic inflammation [22], and it is possible that different intensities of physical activity during specific periods of life (eg: adolescence, premenopause, postmenopause) invoke distinct pathways to influence breast cancer risk.

We previously reported that women in the larger, baseline National Institutes of Health (NIH)-AARP (formerly the "American Association of Retired Persons") Diet and Health Study cohort who were physically active five or more times per week during the year prior to baseline questionnaire administration showed a 13% reduced risk of postmenopausal breast cancer, with suggested heterogeneity in the association according to tumor estrogen receptor (ER) status and lifestyle and reproductive factors [24]. In the present analysis, we followed up these results using detailed information on physical activity from the second stage of the prospective NIH-AARP study to evaluate the association of light and moderate-to-vigorous intensity physical activity during four periods of life from adolescence through adulthood with postmenopausal breast cancer risk overall and by ER status, tumor stage, and histology.

## Methods

### Study population

The NIH-AARP Diet and Health Study [25] was established in 1995-1996, when 3.5 million members of the AARP aged 50 to 71 years who resided in six U.S. states (California, Florida, Louisiana, New Jersey, North Carolina, and Pennsylvania) or two urban areas (Atlanta, Georgia, and Detroit, Michigan) were mailed a baseline questionnaire to assess diet, physical activity, and medical history. The present analysis is based on a detailed second questionnaire sent six months later to baseline questionnaire respondents, which was completed by 334,908 individuals.

After excluding men (n = 196,851), women with prevalent cancers (other than non-melanoma skin cancer) before baseline (n = 8294), those missing all physical activity data from the second questionnaire (n = 1350), those with missing or extreme values of height or weight (the latter defined by the Box-Cox transformation [26], n = 4055), those with no follow-up (n = 6) and women who were premenopausal (n = 4378) or whose menopausal status was unknown before baseline (n = 1075), 118,899 women remained for the current analysis. The Special Studies Institutional Review Board of the National Cancer Institute approved this study, and completion of the self-administered questionnaire was considered to imply informed consent.

### Cohort follow-up and case ascertainment

Participants were followed for change of address by annual linkage to the U.S. Postal Service National Change of Address database, by notifications of undelivered mail, through other address update services, and by direct notice from participants. Vital status was ascertained by annual linkage to the Social Security Administration Death Master File, and searches of the National Death Index (NDI) Plus were used to verify vital status and to provide cause of death information.

Incident breast cancer cases were identified by linkage to eleven state cancer registries, with the addition of Texas, Arizona, and Nevada, to the eight states to which the questionnaires were originally posted. The estimated sensitivity of case ascertainment by cancer registry linkage was previously shown to be 90% [27].

ER status was available from seven (California, Louisiana, Georgia, North Carolina, New Jersey, Arizona, and Nevada) of the eleven reporting states, and tumors were classified as ER-positive at a threshold of at least 10 femtomoles of receptor per milligram total protein. Tumors classified as ER-unknown (n = 1907) included borderline, missing, and unknown ER status.

Breast cancer cases were classified according to tumor histology by using the International Classification of Diseases for Oncology, 3^rd ^edition, coding system, with ductal tumors defined by codes 8500 or 8523, lobular tumors by codes 8520 or 8524, and tumors of mixed ductal-lobular histology by code 8522.

### Physical activity assessment

Physical activity was assessed in the second questionnaire by asking participants to report the duration per week (never, rarely, <1 hour per week, 1-3 hours per week, 4-7 hours per week, >7 hours per week) of light intensity activities given the examples "bowling, golf (riding in a cart), table tennis, slow walking/slow dancing, light calisthenics, light gardening, fishing, horseshoes/croquet, light housework," and of moderate-to-vigorous activities given the examples "tennis, golf (walking), biking, swimming, heavy gardening, weight lifting, basketball/baseball, football/soccer, cheerleading/drill team, handball/racquetball, hiking/climbing mountains, fast walking/fast dancing, rowing, aerobics, jogging/running, heavy housework," during each of four separate life periods (age 15-18 years, 19-29 years, 35-39 years, and in the past 10 years). For each life period across both intensity levels, participants were classified into one of five physical activity categories, with the "never" and "rarely" questionnaire responders collapsed into one "inactive" category. Our physical activity instrument was developed with reference to the Physical Activity Scale for the Elderly (PASE) questionnaire, which has been validated using doubly labeled water (r = 0.58) [28] and has shown high reliability across administrations 3-7 weeks apart (ICC = 0.84) [29].

### Statistical analysis

Cox proportional hazards regression with age as the time metric was used to estimate the relative risk (RR) of breast cancer. Follow-up time was calculated from the scan date of the second questionnaire through the date of diagnosis of breast cancer, death, or the end of the study on December 31, 2003. Models were adjusted for potential confounding variables (Tables 1 &2), and separate analyses were performed with the addition of baseline body mass index (BMI) or BMI at age 18 years and with exclusion of age at menarche as a covariate, since these factors could mediate the relation of physical activity to breast cancer. In secondary analyses, moderate-to-vigorous physical activity was added as a covariate in multivariate models for light physical activity during each period of life, and vice versa. We also assessed potential confounding by history of benign breast disease and by history of mammography in the past three years.

**Table 1:** Characteristics of the study population by light and moderate-to-vigorous intensity physical activity during two periods of life: 118,899 postmenopausal women in the NIH-AARP Diet and Health Study

Light activity	Total	Ages 15-18 years					Ages 19-29 years				
		Never/Rarely	<1 hr/wk	1-3 hrs/wk	4-7 hrs/wk	>7 hrs/wk	Never/Rarely	<1 hr/wk	1-3 hrs/wk	4-7 hrs/wk	>7 hrs/wk
		
Participants, No. (%)	118899	10439 (9)	9199 (8)	26777 (23)	26390 (22)	43700 (37)	5998 (5)	6400 (5)	20987 (18)	27569 (23)	55442 (47)
Age at baseline (yrs)	62.9	62.6	62.4	62.7	62.9	63.1	62.7	62.3	62.5	62.7	63.1
BMI, current* (kg/m^2^)	26.7	26.4	26.3	26.5	26.7	27.0	26.5	26.5	26.6	26.7	26.8
BMI at age 18* (kg/m^2^)	20.9	21.0	21.0	20.9	20.9	20.7	21.0	21.1	21.0	20.9	20.8
Caucasian (%)	91.2	89.5	90.4	91.5	91.9	91.8	85.6	88.4	89.8	91.7	92.9
College education (%)	31.5	32.3	32.9	33.0	33.1	29.7	32.9	34.4	35.7	33.9	28.8
Family history of breast cancer^† ^(%)	13.7	12.8	13.3	13.7	13.8	13.8	13.5	12.8	13.6	13.5	13.8
Current smoker (%)	13.4	11.2	12.2	13.0	12.9	14.7	11.9	11.8	12.8	13.3	14.0
Ever MHT use^‡ ^(%)	61.7	61.2	60.8	61.3	62.0	62.3	60.7	60.3	61.0	61.6	62.5
Age at menarche (yrs)	12.50	12.52	12.49	12.47	12.47	12.52	12.53	12.51	12.48	12.49	12.50
Age at first birth (yrs)	22.98	23.19	23.34	23.24	23.13	22.64	23.19	23.34	23.24	23.13	22.64
Parity (no. of children)	2.01	1.90	1.92	1.94	2.00	2.09	1.87	1.78	1.78	1.90	2.19
Age at menopause(yrs)	47.02	47.31	47.47	47.47	47.35	47.03	47.08	47.43	47.42	47.40	47.14
Alcohol intake (g/d)	6.2	6.5	6.5	6.5	6.3	5.8	6.2	6.3	6.5	6.4	6.0
**Moderate-to-vigorous activity**		**Ages 15-18 years**					**Ages 19-29 years**				
		Never/Rarely	<1 hr/wk	1-3 hrs/wk	4-7 hrs/wk	>7 hrs/wk	Never/Rarely	<1 hr/wk	1-3 hrs/wk	4-7 hrs/wk	>7 hrs/wk
		
Participants, No. (%)	118899	15359 (13)	10080 (9)	25250 (21)	26429 (22)	39845 (34)	11585 (10)	9867 (8)	25686 (22)	30175 (25)	39603 (33)
Age at baseline (yrs)	62.9	62.9	62.8	62.9	62.8	62.8	62.8	62.5	62.6	62.8	63.2
BMI, current* (kg/m^2^)	26.7	26.7	26.6	26.6	26.6	26.9	27.0	26.8	26.6	26.6	26.8
BMI at age 18* (kg/m^2^)	20.9	21.3	21.0	21.0	20.8	20.7	21.3	21.1	20.9	20.8	20.7
Caucasian (%)	91.2	90.4	91.1	91.4	92.0	91.6	88.8	90.6	91.1	92.1	92.3
College education (%)	31.5	30.5	31.5	32.4	33.7	30.7	33.7	36.3	35.3	32.8	27.0
Family history of breast cancer^† ^(%)	13.7	13.5	12.7	13.7	14.0	13.7	13.4	13.0	13.8	13.6	13.9
Current smoker (%)	13.4	10.1	10.9	12.7	13.4	15.7	10.6	11.2	12.0	13.4	15.7
Ever MHT use^‡ ^(%)	61.7	59.2	60.9	61.5	63.3	62.4	58.4	62.1	62.1	62.9	61.8
Age at menarche (yrs)	12.50	12.42	12.45	12.48	12.50	12.55	12.41	12.46	12.49	12.50	12.54
Age at first birth (yrs)	22.98	23.32	23.26	23.18	23.03	22.66	23.56	23.54	23.40	23.04	22.45
Parity (no. of children)	2.01	1.86	1.94	1.97	2.02	2.09	1.75	1.82	1.88	2.02	2.20
Age at menopause(yrs)	47.02	47.43	47.52	47.46	47.30	46.99	47.49	47.64	47.54	47.30	46.96
Alcohol intake (g/d)	6.2	6.0	6.2	6.3	6.4	6.1	6.0	6.1	6.3	6.3	6.1

*BMI: body mass index.

†Family history of breast cancer in a first-degree female relative.

‡MHT: menopausal hormone therapy.

**Table 2:** Characteristics of the study population by light and moderate-to-vigorous intensity physical activity during two periods of life: 118,899 postmenopausal women in the NIH-AARP Diet and Health Study

Light activity	Total	Ages 35-39 years					Past 10 years				
		Never/Rarely	<1 hr/wk	1-3 hrs/wk	4-7 hrs/wk	>7 hrs/wk	Never/Rarely	<1 hr/wk	1-3 hrs/wk	4-7 hrs/wk	>7 hrs/wk
		
Participants, No. (%)	118899	5189 (4)	6121 (5)	20710 (17)	29018 (24)	55602 (47)	5789 (5)	6811 (6)	24450 (21)	32432 (27)	47827 (40)
Age at baseline (yrs)	62.9	62.6	62.2	62.4	62.6	63.3	62.5	62.3	62.4	62.9	63.2
BMI, current* (kg/m^2^)	26.7	27.0	27.3	26.8	26.7	26.6	28.6	28.4	27.4	26.6	26.0
BMI at age 18* (kg/m^2^)	20.9	21.1	21.1	21.0	20.9	20.8	21.3	21.1	21.0	20.8	20.8
Caucasian (%)	91.2	85.3	85.8	89.5	91.8	93.1	84.9	85.0	90.0	92.3	93.1
College education (%)	31.5	31.8	33.6	35.0	34.3	28.9	27.5	29.8	34.0	33.8	29.6
Family history of breast cancer^† ^(%)	13.7	13.1	13.8	13.4	13.5	13.9	13.6	13.3	13.4	13.6	13.8
Current smoker (%)	13.4	12.6	12.8	13.3	13.1	13.7	16.7	15.1	13.7	12.9	12.9
Ever MHT use^‡ ^(%)	61.7	59.0	58.7	61.6	62.5	62.1	56.9	58.3	61.8	62.8	62.0
Age at menarche (yrs)	12.50	12.51	12.48	12.48	12.48	12.51	12.48	12.47	12.47	12.48	12.53
Age at first birth (yrs)	22.98	22.98	23.33	23.30	23.23	22.73	22.75	23.04	23.20	23.14	22.81
Parity (no. of children)	2.01	1.87	1.79	1.77	1.90	2.19	1.92	1.88	1.89	1.98	2.11
Age at menopause(yrs)	47.02	47.01	47.31	47.32	47.34	47.24	46.59	47.00	47.20	47.43	47.34
Alcohol intake (g/d)	6.2	6.0	6.1	6.3	6.4	6.1	6.1	5.5	6.1	6.3	6.2
**Moderate-to-vigorous activity**		**Ages 35-39 years**					**Past 10 years**				
		Never/Rarely	<1 hr/wk	1-3 hrs/wk	4-7 hrs/wk	>7 hrs/wk	Never/Rarely	<1 hr/wk	1-3 hrs/wk	4-7 hrs/wk	>7 hrs/wk
		
Participants, No. (%)	118899	10868 (9)	10507 (9)	26715 (23)	31560 (27)	37477 (32)	17028 (14)	12498 (11)	29927 (25)	30231 (25)	28214 (24)
Age at baseline (yrs)	62.9	62.8	62.5	62.5	62.7	63.4	62.9	62.5	62.7	62.9	63.1
BMI, current* (kg/m^2^)	26.7	27.6	27.3	26.8	26.5	26.4	28.7	28.0	26.9	26.0	25.4
BMI at age 18* (kg/m^2^)	20.9	21.3	21.0	20.9	20.8	20.7	21.2	21.0	20.8	20.8	20.7
Caucasian (%)	91.2	88.0	89.1	90.9	92.6	92.6	88.7	89.7	91.4	92.7	92.4
College education (%)	31.5	33.2	35.8	35.7	33.0	26.4	30.3	31.8	33.7	33.7	27.9
Family history of breast cancer^† ^(%)	13.7	13.0	13.4	13.7	13.6	14.0	13.3	13.9	13.4	14.1	13.6
Current smoker (%)	13.4	12.0	12.3	12.5	13.3	14.8	15.5	14.3	13.6	12.6	12.5
Ever MHT use^‡ ^(%)	61.7	56.7	60.1	63.0	63.3	61.8	57.8	60.8	62.1	64.1	61.6
Age at menarche (yrs)	12.50	12.38	12.48	12.48	12.49	12.55	12.40	12.46	12.48	12.52	12.56
Age at first birth (yrs)	22.98	23.39	23.40	23.25	23.02	22.60	23.06	23.06	23.07	23.06	22.76
Parity (no. of children)	2.01	1.74	1.81	1.89	2.02	2.22	1.88	1.95	1.99	2.04	2.09
Age at menopause(yrs)	47.02	47.39	47.42	47.42	47.33	47.05	46.94	47.17	47.34	47.40	47.26
Alcohol intake (g/d)	6.2	5.7	5.9	6.3	6.5	6.2	5.8	5.8	6.1	6.4	6.5

*BMI: body mass index.

†Family history of breast cancer in a first-degree female relative.

‡MHT: menopausal hormone therapy.

Stratified analyses were conducted to explore variation in the physical activity-breast cancer association by previously reported effect modifiers including baseline BMI, BMI at age 18 years, family history of breast cancer in first-degree female relatives, parity, and menopausal hormone therapy use, and the statistical significance of the interaction was assessed by likelihood ratio tests comparing models with and without interaction terms.

In separate analyses, we investigated the relation of physical activity with breast cancer endpoint subgroups defined by ER status (ER-negative, ER-positive, or ER-unknown tumors), tumor stage (invasive or *in situ *breast cancer), and histological subtype (ductal, lobular, or mixed ductal-lobular histology). Analyses of ER status and tumor histology were restricted to invasive cancers. Heterogeneity by the above tumor characteristics was evaluated using Cochran's Q statistic comparing risk estimates for the highest versus lowest physical activity level, for each distinct endpoint [30].

Spearman rank correlation coefficients (ρ) examined the inter-relationship of physical activity measures across categories of intensity and time of life.

Analyses were performed using SAS (version 9.1; SAS Institute, Inc., Cary, North Carolina), with all statistical tests two-sided and conducted at the 0.05 significance level.

## Results

Women were, on average, 62.9 years old at baseline, and most women were Caucasian (91.2%) and 31.5% were college-educated (Tables 1 &2). Nearly fifty percent of participants reported engaging in more than three hours of moderate-to-vigorous physical activity during an average week in the past ten years (Table 2). Light intensity activities were more prevalent than moderate-to-vigorous activities across the lifespan, and women reported spending more time in moderate-to-vigorous activities during the younger life periods than during the past ten years.

Women who reported a high level of light or moderate-to-vigorous activity between ages 15-18 years had a slightly higher baseline BMI than women who were inactive at ages 15-18 years, whereas women who reported being highly active from ages 35-39 years and during the 10 years prior to questionnaire administration tended to have a lower baseline BMI than their inactive counterparts. Regardless of intensity level and time of life of activity, the most active women reported that they were leaner at age 18 years than inactive women. Women with a high level of physical activity in the past 10 years were less likely to currently smoke cigarettes and more likely to drink alcohol than inactive women; however, compared with less active women, those with the highest level of activity from ages 15-18, 19-29 or 35-39 years were more likely to be current cigarette smokers.

Positive correlations were observed between light and moderate-to-vigorous intensity activity within the same periods of life (ρ = 0.49-0.53) (Table 3). Regardless of intensity level, correlations were modest between physical activity in the past 10 years and activity from ages 15-18 years (ρ = 0.35 for light activity, ρ = 0.28 for moderate-to-vigorous activity), but were stronger for less distant life periods of activity such as ages 19-29 and 35-39 years (ρ = 0.79 for both light and moderate-to-vigorous activity).

**Table 3:** Spearman correlation coefficients for the relationship among physical activity variables

Physical activity, by intensity and period of life	Physical activity, by intensity and period of life						
	Light physical activity			Moderate-to-vigorous physical activity			
**Light physical activity**	15-18 years	19-29 years	35-39 years	15-18 years	19-29 years	35-39 years	Past 10 years
15-18 years	-	0.65	0.52	0.53	0.41	0.30	0.12
19-29 years	0.65	-	0.79	0.38	0.50	0.42	0.21
35-39 years	0.52	0.79	-	0.31	0.44	0.49	0.29
Past 10 years	0.35	0.53	0.66	0.21	0.32	0.39	0.49
	**Moderate-to-vigorous physical activity**						
**Moderate-to-vigorous physical activity**	15-18 years	19-29 years	35-39 years				
15-18 years	-	0.69	0.53				
19-29 years	0.69	-	0.79				
35-39 years	0.53	0.79	-				
Past 10 years	0.28	0.45	0.61				

Over 6.6 years of follow-up, 4287 incident breast cancers were diagnosed (including 736 *in situ *tumors). The majority of tumors with known ER status were ER-positive (84%, n = 1352). Women diagnosed with incident breast cancer were more likely than those who were not diagnosed with breast cancer to have a first-degree female relative with breast cancer and to report an earlier age at menarche, later age at first birth, lower parity, later age at menopause, slightly higher BMI, increased alcohol intake and to have ever used MHT (data not shown).

We did not observe an association of physical activity from ages 15-18, 19-29, or 35-39 years with postmenopausal breast cancer risk, regardless of intensity level (Tables 4 &5). Physical activity of moderate-to-vigorous intensity during the past 10 years was statistically significantly associated with reduced risk of postmenopausal breast cancer (Table 5). Specifically, women engaging in >7 hours per week of moderate-to-vigorous activity showed a 16% reduced breast cancer risk as compared with their inactive counterparts in both age-adjusted (RR: 0.84, 95%CI:0.76,0.93) and multivariate (RR:0.84, 95%CI:0.76,0.93) analyses. Additional adjustment for light intensity activity during the same time period did not substantially alter risk estimates (RR: 0.85, 95%CI:0.76,0.95), and adjustment for current BMI slightly attenuated but did not abolish the multivariate association (RR:0.87, 95%CI:0.78,0.96).

**Table 4:** Relative risk (RR) and 95% confidence intervals (CI) for the association of light physical activity during four periods of life with breast cancer incidence: 118,899 postmenopausal women in the NIH-AARP Diet and Health Study

Light activity	No. cancers* n = 4287	**RR**^†^	95% CI	**RR**^††^	95% CI		**RR**^§^	95% CI		**RR**^£^	95% CI	
Ages 15-18 years												
Never/Rarely	358	1.00 (ref.)		1.00 (ref.)			1.00 (ref.)			1.00 (ref.)		
<1 hour/week	344	1.09	0.94, 1.27	1.09	0.94	1.26	1.07	0.92	1.24	1.09	0.94	1.26
1-3 hours/week	974	1.06	0.94, 1.20	1.05	0.93	1.19	1.04	0.92	1.18	1.05	0.93	1.18
4-7 hours/week	982	1.08	0.96, 1.22	1.08	0.95	1.22	1.07	0.95	1.22	1.07	0.95	1.21
>7 hours/week	1553	1.03	0.92, 1.16	1.05	0.94	1.18	1.05	0.93	1.19	1.04	0.93	1.17
												
Ages 19-29 years												
Never/Rarely	199	1.00 (ref.)		1.00 (ref.)			1.00 (ref.)			1.00 (ref.)		
<1 hour/week	249	1.18	0.98, 1.42	1.16	0.97	1.40	1.15	0.95	1.38	1.16	0.96	1.40
1-3 hours/week	793	1.14	0.98, 1.34	1.11	0.95	1.30	1.10	0.94	1.29	1.11	0.95	1.30
4-7 hours/week	1036	1.13	0.97, 1.32	1.12	0.96	1.30	1.11	0.95	1.30	1.11	0.96	1.30
>7 hours/week	1940	1.05	0.91, 1.21	1.07	0.93	1.24	1.07	0.92	1.25	1.07	0.92	1.24
												
Ages 35-39 years												
Never/Rarely	171	1.00 (ref.)		1.00 (ref.)			1.00 (ref.)			1.00 (ref.)		
<1 hour/week	238	1.19	0.98, 1.44	1.17	0.96	1.42	1.14	0.94	1.40	1.16	0.95	1.41
1-3 hours/week	811	1.19	1.01, 1.41	1.15	0.98	1.36	1.15	0.97	1.36	1.15	0.98	1.36
4-7 hours/week	1036	1.08	0.92, 1.27	1.05	0.89	1.24	1.04	0.88	1.23	1.05	0.91	1.24
>7 hours/week	1952	1.05	0.90, 1.23	1.06	0.90	1.24	1.06	0.90	1.24	1.06	0.91	1.24
												
Past 10 years												
Never/Rarely	183	1.00 (ref.)		1.00 (ref.)			1.00 (ref.)			1.00 (ref.)		
<1 hour/week	266	1.23	1.02, 1.48	1.21	1.00	1.46	1.19	0.98	1.44	1.21	1.00	1.46
1-3 hours/week	890	1.13	0.96, 1.32	1.08	0.92	1.27	1.10	0.93	1.29	1.09	0.93	1.28
4-7 hours/week	1264	1.20	1.02, 1.40	1.14	0.98	1.34	1.19	1.01	1.39	1.16	0.99	1.36
>7 hours/week	1634	1.04	0.89, 1.21	1.01	0.87	1.18	1.09	0.92	1.28	1.04	0.89	1.21

* Number of cancer cases may not add up to total (n = 4287) due to missing physical activity values

† Relative risks adjusted for age (continuous)

†† Relative risks adjusted for: age (continuous); race/ethnicity (white, black, Hispanic, Asian/Pacific Islander/American Indian); education level (<12 years or high school equivalent, 12 years or high school equivalent, post-high school vocational or technical training, some college, college graduate, post-graduate); smoking status (non-smoker, former, current); alcohol intake (grams/day, quintiles adjusted for total energy intake); family history of breast cancer (no, yes); age at menarche (<13, 13-14, ≥ 15 years); age at first birth (nulliparous, <20, 20-24, 25-29, 30+ years); parity (number of children: 0, 1-2, 3 or more); age at menopause (<40, 40-44, 45-49, 50-54, ≥ 55 years); menopausal hormone use (never, ever)

§Relative risks additionally adjusted for frequency of other type of physical activity (either light or moderate-to-vigorous) during that same time period

£ Relative risks additionally adjusted for body mass index (BMI: <25.0, 25.0-29.9, 30.0-34.9, ≥ 35.0 kg/m^2^)

**Table 5:** Relative risk (RR) and 95% confidence intervals (CI) for the association of moderate-to-vigorous physical activity during four periods of life with breast cancer incidence: 118,899 postmenopausal women in the NIH-AARP Diet and Health Study

Moderate-to-vigorous activity	No. cancers* n = 4287	**RR**^†^	95% CI	**RR**^††^	95% CI		**RR**^§^	95% CI		**RR**^£^	95% CI	
Ages 15-18 years												
Never/Rarely	541	1.00 (ref.)		1.00 (ref.)			1.00 (ref.)			1.00 (ref.)		
<1 hour/week	394	1.11	0.98, 1.27	1.12	0.98	1.27	1.10	0.97	1.26	1.12	0.98	1.27
1-3 hours/week	936	1.05	0.95, 1.17	1.05	0.94	1.17	1.04	0.93	1.16	1.05	0.94	1.16
4-7 hours/week	950	1.02	0.92, 1.14	1.01	0.91	1.13	1.00	0.89	1.12	1.01	0.91	1.13
>7 hours/week	1415	1.02	0.92, 1.12	1.03	0.93	1.14	1.02	0.91	1.14	1.02	0.93	1.13
												
Ages 19-29 years												
Never/Rarely	415	1.00 (ref.)		1.00 (ref.)			1.00 (ref.)			1.00 (ref.)		
<1 hour/week	384	1.09	0.95, 1.25	1.08	0.94	1.24	1.07	0.93	1.23	1.08	0.94	1.24
1-3 hours/week	976	1.07	0.95, 1.20	1.06	0.94	1.19	1.05	0.93	1.18	1.06	0.94	1.19
4-7 hours/week	1080	1.00	0.89, 1.12	1.01	0.90	1.13	1.01	0.89	1.13	1.01	0.90	1.13
>7 hours/week	1375	0.97	0.87, 1.08	1.01	0.90	1.13	1.02	0.90	1.15	1.01	0.90	1.13
												
Ages 35-39 years												
Never/Rarely	392	1.00 (ref.)		1.00 (ref.)			1.00 (ref.)			1.00 (ref.)		
<1 hour/week	421	1.12	0.98, 1.28	1.11	0.97	1.27	1.09	0.95	1.25	1.11	0.97	1.27
1-3 hours/week	963	1.00	0.89, 1.13	0.99	0.88	1.11	0.98	0.87	1.11	0.99	0.88	1.12
4-7 hours/week	1174	1.03	0.92, 1.16	1.03	0.92	1.15	1.04	0.92	1.18	1.04	0.93	1.16
>7 hours/week	1288	0.94	0.84, 1.06	0.97	0.87	1.09	0.99	0.87	1.12	0.98	0.88	1.10
												
Past 10 years												
Never/Rarely	646	1.00 (ref.)		1.00 (ref.)			1.00 (ref.)			1.00 (ref.)		
<1 hour/week	507	1.06	0.94, 1.19	1.05	0.94	1.18	1.03	0.92	1.16	1.06	0.94	1.19
1-3 hours/week	1071	0.93	0.84, 1.02	0.91	0.83	1.00	0.90	0.81	1.00	0.93	0.84	1.02
4-7 hours/week	1106	0.94	0.86, 1.04	0.92	0.83	1.01	0.91	0.82	1.01	0.94	0.85	1.04
>7 hours/week	928	0.84	0.76, 0.93	0.84	0.76	0.93	0.85	0.76	0.95	0.87	0.78	0.96

Number of cancer cases may not add up to total (n = 4287) due to missing physical activity values

† Relative risks adjusted for age (continuous)

†† Relative risks adjusted for: age (continuous); race/ethnicity (white, black, Hispanic, Asian/Pacific Islander/American Indian); education level (<12 years or high school equivalent, 12 years or high school equivalent, post-high school vocational or technical training, some college, college graduate, post-graduate); smoking status (non-smoker, former, current); alcohol intake (grams/day, quintiles adjusted for total energy intake); family history of breast cancer (no, yes); age at menarche (<13, 13-14, ≥ 15 years); age at first birth (nulliparous, <20, 20-24, 25-29, 30+ years); parity (number of children: 0, 1-2, 3 or more); age at menopause (<40, 40-44, 45-49, 50-54, ≥ 55 years); menopausal hormone use (never, ever)

§Relative risks additionally adjusted for frequency of other type of physical activity (either light or moderate-to-vigorous) during that same time period

£ Relative risks additionally adjusted for body mass index (BMI: <25.0, 25.0-29.9, 30.0-34.9, ≥ 35.0 kg/m^2^)

Including variables representing BMI at age 18 years, history of mammography, previous benign breast disease, or excluding age at menarche in serial multivariate models did not change risk estimates. No statistically significant heterogeneity in the association of physical activity with breast cancer risk was observed across strata of baseline BMI, BMI at age 18 years, family history of breast cancer, parity, or menopausal hormone therapy use (data not shown).

Physical activity from ages 15-18, 19-29 and 35-39 years was not associated with breast cancer tumor characteristics (Tables 6 &7). Results are shown for multivariate models without BMI, as addition of BMI to statistical models did not substantially alter risk estimates. More than 7 hours per week of moderate-to-vigorous physical activity during the past 10 years was statistically significantly associated with reduced risk of ER-positive tumors (Table 7, RR:0.77, 95%CI:0.64,0.92). The observed relationship with ER-negative tumors for >7 hours per week of moderate-to-vigorous activity during the past 10 years was also inverse (RR: 0.87, 95%CI:0.58,1.29), but failed to reach statistical significance. Inverse associations with moderate-to-vigorous activity in the past ten years were of similar magnitude for both invasive (RR:0.84, 95%CI:0.75,0.94) and *in situ *(RR:0.85, 95%CI:0.66,1.08) tumors, and associations did not vary by tumor histology (data not shown).

**Table 6:** Multivariate adjusted relative risk (RR)* and 95% confidence intervals (CI) for the association of light physical activity during four periods of life with breast cancer tumor characteristics: 118,899 postmenopausal women in the NIH-AARP Diet and Health Study

Light activity	ER- positive ^†^**RR**	95% CI		ER- negative RR	95% CI		**p**^††^	ER- unknown RR	95% CI		**p**^§^	Invasive RR	95% CI		In situ RR	95% CI		**p**^£^
Ages 15-18 years																		
Never/Rarely	1.00 (ref.)			1.00 (ref.)				1.00 (ref.)				1.00 (ref.)			1.00 (ref.)			
<1 hour/week	0.99	0.75	1.29	1.03	0.56	1.90		1.28	1.03	1.60		1.14	0.97	1.35	0.90	0.63	1.27	
1-3 hours/week	0.97	0.78	1.21	1.07	0.65	1.74		1.10	0.91	1.33		1.04	0.91	1.20	1.06	0.81	1.39	
4-7 hours/week	1.06	0.86	1.31	1.11	0.68	1.81		1.13	0.94	1.36		1.10	0.96	1.26	0.99	0.75	1.30	
>7 hours/week	1.09	0.89	1.33	1.02	0.64	1.62	0.79	1.13	0.95	1.35	0.90	1.10	0.97	1.26	0.81	0.62	1.06	0.03
																		
Ages 19-29 years																		
Never/Rarely	1.00 (ref.)			1.00 (ref.)				1.00 (ref.)				1.00 (ref.)			1.00 (ref.)			
<1 hour/week	1.20	0.88	1.66	0.88	0.41	1.87		1.09	0.82	1.46		1.12	0.91	1.37	1.48	0.94	2.32	
1-3 hours/week	1.11	0.85	1.45	0.91	0.50	1.66		1.05	0.83	1.33		1.06	0.89	1.26	1.38	0.94	2.04	
4-7 hours/week	1.00	0.77	1.31	1.04	0.59	1.86		1.17	0.93	1.48		1.09	0.92	1.29	1.31	0.90	1.92	
>7 hours/week	0.99	0.77	1.27	1.04	0.59	1.86	0.99	1.14	0.91	1.42	0.00	1.06	0.90	1.25	1.15	0.79	1.66	0.91
																		
Ages 35-39 years																		
Never/Rarely	1.00 (ref.)			1.00 (ref.)				1.00 (ref.)				1.00 (ref.)			1.00 (ref.)			
<1 hour/week	1.07	0.77	1.50	1.14	0.46	2.84		1.21	0.90	1.65		1.15	0.92	1.43	1.26	0.80	1.99	
1-3 hours/week	1.07	0.81	1.41	1.48	0.70	3.13		1.17	0.91	1.52		1.15	0.95	1.38	1.17	0.80	1.73	
4-7 hours/week	0.91	0.69	1.19	1.44	0.69	3.00		1.12	0.87	1.44		1.04	0.87	1.25	1.07	0.73	1.57	
>7 hours/week	0.89	0.68	1.16	1.48	0.72	3.02	0.20	1.20	0.94	1.53	0.18	1.08	0.91	1.28	0.94	0.65	1.37	0.52
																		
Past 10 years																		
Never/Rarely	1.00 (ref.)			1.00 (ref.)				1.00 (ref.)				1.00 (ref.)			1.00 (ref.)			
<1 hour/week	1.20	0.86	1.69	0.35	0.14	0.92		1.24	0.94	1.64		1.14	0.93	1.41	1.50	0.95	2.38	
1-3 hours/week	1.06	0.80	1.41	1.04	0.59	1.86		1.00	0.79	1.27		1.03	0.86	1.22	1.35	0.91	2.02	
4-7 hours/week	1.19	0.90	1.57	0.94	0.53	1.66		1.11	0.88	1.40		1.12	0.95	1.33	1.24	0.83	1.84	
>7 hours/week	0.92	0.70	1.22	0.80	0.45	1.40	0.65	1.07	0.85	1.35	0.53	0.99	0.84	1.17	1.11	0.75	1.63	0.61

No. cancer cases**	1352			263				1907				3522			736			

* Relative risks adjusted for: age (continuous); race/ethnicity (white, black, Hispanic, Asian/Pacific Islander/American Indian); education level (<12 years or high school equivalent, 12 years or high school equivalent, post-high school vocational or technical training, some college, college graduate, post-graduate); smoking status (non-smoker, former, current); alcohol intake (grams/day, quintiles adjusted for total energy intake); family history of breast cancer (no, yes); age at menarche (<13, 13-14, ≥ 15 years); age at first birth (nulliparous, <20, 20-24, 25-29, 30+ years); parity (number of children: 0, 1-2, 3 or more); age at menopause (<40, 40-44, 45-49, 50-54, ≥ 55 years); menopausal hormone use (never, ever)

** Total numbers of cancer cases may vary due to missing physical activity values.

† ER: estrogen receptor. Hormone receptor status limited to invasive cancers.

†† p-value for heterogeneity comparing RR for >7 hours/week of physical activity: ER-positive and ER-negative tumors.

§p-value for heterogeneity comparing RR for >7 hours/week of physical activity: ER-positive, ER-negative, and ER-unknown tumors.

£ p-value for heterogeneity comparing RR for >7 hours/week of physical activity: invasive and *in situ *tumors

**Table 7:** Multivariate adjusted relative risk (RR)* and 95% confidence intervals (CI) for the association of light physical activity during four periods of life with breast cancer tumor characteristics: 118,899 postmenopausal women in the NIH-AARP Diet and Health Study

Moderate/vigorous activity	ER-positive ^†^**RR**	95% CI		ER- negative RR	95% CI		**p**^††^	ER- unknown RR	95% CI		**p**^§^	Invasive RR	95% CI		In situ RR	95% CI		**p**^£^
Ages 15-18 years																		
Never/Rarely	1.00 (ref.)			1.00 (ref.)				1.00 (ref.)				1.00 (ref.)			1.00 (ref.)			
<1 hour/week	1.05	0.82	1.34	0.85	0.48	1.49		1.08	0.89	1.31		1.05	0.91	1.22	1.40	1.04	1.87	
1-3 hours/week	1.10	0.90	1.34	1.11	0.73	1.68		0.96	0.82	1.13		1.02	0.91	1.15	1.20	0.93	1.53	
4-7 hours/week	1.22	1.00	1.47	0.98	0.64	1.50		0.94	0.80	1.10		1.04	0.93	1.17	0.90	0.70	1.17	
>7 hours/week	1.18	0.98	1.42	0.91	0.61	1.36	0.24	1.02	0.88	1.18	0.33	1.06	0.95	1.19	0.89	0.70	1.17	0.18
																		
Ages 19-29 years																		
Never/Rarely	1.00 (ref.)			1.00 (ref.)				1.00 (ref.)				1.00 (ref.)			1.00 (ref.)			
<1 hour/week	1.18	0.92	1.52	0.99	0.56	1.76		1.01	0.82	1.24		1.07	0.92	1.25	1.12	0.81	1.54	
1-3 hours/week	1.14	0.92	1.41	0.90	0.56	1.44		0.99	0.84	1.18		1.04	0.91	1.18	1.14	0.87	1.49	
4-7 hours/week	1.16	0.94	1.43	1.15	0.74	1.80		0.94	0.79	1.11		1.03	0.91	1.17	0.94	0.71	1.23	
>7 hours/week	1.14	0.93	1.40	0.96	0.61	1.49	0.48	0.99	0.84	1.16	0.51	1.04	0.92	1.18	0.87	0.67	1.13	0.23
																		
Ages 35-39 years																		
Never/Rarely	1.00 (ref.)			1.00 (ref.)				1.00 (ref.)				1.00 (ref.)			1.00 (ref.)			
<1 hour/week	1.17	0.91	1.49	0.93	0.51	1.71		1.03	0.83	1.27		1.07	0.92	1.25	1.23	0.90	1.68	
1-3 hours/week	0.99	0.80	1.23	1.25	0.77	2.02		0.95	0.79	1.13		0.99	0.86	1.12	0.99	0.75	1.30	
4-7 hours/week	1.08	0.88	1.33	1.14	0.71	1.84		1.03	0.87	1.22		1.06	0.93	1.20	0.92	0.70	1.21	
>7 hours/week	1.01	0.82	1.24	1.01	0.63	1.63	0.99	0.99	0.83	1.17	0.99	1.00	0.88	1.13	0.85	0.65	1.12	0.31
																		
Past 10 years																		
Never/Rarely	1.00 (ref.)			1.00 (ref.)				1.00 (ref.)				1.00 (ref.)			1.00 (ref.)			
<1 hour/week	1.18	0.97	1.45	0.60	0.35	1.03		1.00	0.84	1.19		1.04	0.91	1.18	1.12	0.85	1.49	
1-3 hours/week	0.96	0.80	1.14	0.79	0.53	1.17		0.88	0.76	1.01		0.90	0.81	1.00	0.99	0.78	1.25	
4-7 hours/week	0.98	0.83	1.17	0.99	0.68	1.45		0.87	0.75	1.01		0.92	0.83	1.03	0.90	0.71	1.14	
>7 hours/week	0.77	0.64	0.92	0.87	0.58	1.29	0.58	0.89	0.77	1.03	0.56	0.84	0.75	0.94	0.85	0.66	1.08	0.96

No. cancer cases**	1352			263				1907				3522			736			

* Relative risks adjusted for: age (continuous); race/ethnicity (white, black, Hispanic, Asian/Pacific Islander/American Indian); education level (<12 years or high school equivalent, 12 years or high school equivalent, post-high school vocational or technical training, some college, college graduate, post-graduate); smoking status (non-smoker, former, current); alcohol intake (grams/day, quintiles adjusted for total energy intake); family history of breast cancer (no, yes); age at menarche (<13, 13-14, ≥ 15 years); age at first birth (nulliparous, <20, 20-24, 25-29, 30+ years); parity (number of children: 0, 1-2, 3 or more); age at menopause (<40, 40-44, 45-49, 50-54, ≥ 55 years); menopausal hormone use (never, ever)

** Total numbers of cancer cases may vary due to missing physical activity values.

† ER: estrogen receptor. Hormone receptor status limited to invasive cancers.

†† p-value for heterogeneity comparing RR for >7 hours/week of physical activity: ER-positive and ER-negative tumors.

§p-value for heterogeneity comparing RR for >7 hours/week of physical activity: ER-positive, ER-negative, and ER-unknown tumors.

£ p-value for heterogeneity comparing RR for >7 hours/week of physical activity: invasive and *in situ *tumors

## Discussion

We observed that >7 hour per week of recent (in the past 10 years) but not historical (from ages 15-18, 19-29, or 35-39 years) moderate-to-vigorous intensity physical activity was associated with a 16% reduced risk of postmenopausal breast cancer. No relation between breast cancer risk and light intensity physical activity during any of the periods of life examined was observed. Associations did not appear to vary by ER status, breast tumor stage, or tumor histological subtype.

The inverse association observed in our study (RR:0.84, 95%CI:0.76,0.93) is similar to the 13% reduction in postmenopausal breast cancer risk (RR:0.87, 95%CI:0.81,0.95) observed for women reporting ≥ 5 episodes per week of physical activity during the year preceding assessment in the baseline NIH-AARP cohort [24]. Although both of these risk estimates are less pronounced than the 20-40% risk reductions reported in reviews of the physical activity-breast cancer relationship [3,5], directly comparing risk estimates across studies is difficult considering variation in the instruments used to measure physical activity, inconsistencies in the parameters of physical activity that are assessed, and underlying differences in the study populations.

Only two previous studies [7,14] reported on both the intensity level and time of life of physical activity in relation to breast cancer risk. A Polish case-control study [14] evaluated the association of physical activity of various intensities from light to vigorous across decades of life (age 20-24, 25-29, 30-34, 35-39, 40-49, 50-59, 60-69 years). Significant inverse associations were observed for physical activity across multiple decades of life with risk of breast cancer. Inverse associations were generally stronger for moderate-to-vigorous recreational activity than for total recreational activity, and a high level of moderate-to-vigorous recreational activity during ages 50-59 years was associated with the largest reduction in breast cancer risk.

In the second study to look at the combination of intensity and time in life of physical activity [7], the California Teachers' Study (CTS) investigated moderate and strenuous intensity physical activity from ages 18-24, 25-34, 35-44, 45-54 years and in the 3 years preceding baseline assessment. Contrasting the Polish case-control study [14] and our results, the CTS [7] observed no association of moderate or strenuous physical activity during the three years preceding baseline with breast cancer risk. Yet a high versus low level of strenuous physical activity from age 18 years through the three years preceding baseline was associated with a 20% reduced risk of breast cancer (RR:0.80, 95%CI:0.69-0.94), and moderate physical activity from age 18 years through the three years preceding baseline showed a statistically non-significant 6% reduced breast cancer risk (RR:0.94, 95%CI:0.81-1.08) [7]. As the relation of physical activity with breast cancer risk appears to vary by menopausal status [5], inconsistent results may be due to differences in the study populations; whereas our study included only postmenopausal women, and the Polish case-control study was comprised of 70.5% postmenopausal women, only 51.4% of women in the CTS were postmenopausal.

Our observation that recent physical activity showed a stronger inverse association with breast cancer risk than historical activity is supported by two systematic reviews [3,5] and three prospective studies among postmenopausal women [12,19,20]. Furthermore, our finding that moderate-to-vigorous but not light physical activity was inversely related to breast cancer risk is supported by some [9,14,19], but not all [10,13], studies on the topic. Many previous reports of the relation of physical activity intensity with breast cancer risk have compared moderate with vigorous physical activity; numerous studies [5-9,19] have shown stronger associations for vigorous intensity activity, but other studies have not found a dose-response according to physical activity intensity [10-18]. The physical activity questionnaire in our study combined moderate and vigorous intensity categories, which is consistent with the intensity level cited in the current public health recommendations [31,32]. However, we could not evaluate heterogeneity in the association of moderate versus vigorous activity with breast cancer risk within our study in order to compare with prior studies or to determine whether moderate activity is sufficient for reducing postmenopausal breast cancer risk.

The observed protective effect of moderate-to-vigorous physical activity in our study is biologically plausible, as there is some evidence that a high intensity of physical activity is associated with a reduction in estradiol [33] and enhanced sensitivity to insulin [34]. Whereas recent studies suggest that in postmenopausal women the effect of physical activity on estrogens may be mediated by loss of body fat [35,36], the lack of confounding by or interaction with BMI in our study supports an independent influence of physical activity. Physical activity also appears to modulate immunity in a manner in which moderate activity is beneficial to the immune response, but sedentary behavior and highly strenuous activity can impair immune function [37].

We observed very little heterogeneity in the relation of physical activity to breast cancer tumor characteristics, regardless of the intensity or time of life of physical activity. A lack of heterogeneity in the relationship of physical activity with ER-defined breast cancer is consistent with the Breast Cancer Detection Demonstration Project cohort study [6] and five case-control studies [14,15,38-40]. However, other studies have reported stronger inverse associations of physical activity with ER-positive tumors [14,41,42] or ER-negative tumors [5,7,24]. A suggested inverse relation of recent moderate-to-vigorous activity with ER-negative tumors was observed in both the current study and the baseline NIH-AARP cohort [24]. Yet the substantially smaller number of ER-negative breast cancer cases in the present study likely limited our ability to detect heterogeneity by ER-status.

In addition, we observed similar associations for recent moderate-to-vigorous physical activity with invasive and *in situ *breast cancer. This finding is consistent with three cohort studies [7,12,24] and four case-control studies [14,38,43,44], but diverges somewhat from the Cancer Prevention Study II (CPSII) [20] and a German case-control study [41] in which no association was apparent between recent recreational physical activity and *in situ *breast cancer. However, each of these studies [20,41] included only 205 *in situ *tumors and thus may have lacked power to detect an association.

Observation of similar associations of moderate-to-vigorous activity during the past 10 years with ductal and lobular histological subtypes in our study corresponds to results from a Polish case-control study [14]. These results suggest that moderate-to-vigorous intensity physical activity influences postmenopausal breast cancer risk through estrogenic and non-estrogenic pathways, in both early and more advanced tumors, and affects tumors of a variety of histological subtypes.

Strengths of our study include the large sample size, the prospective design, and the information on timing and intensity of physical activity. In addition, with the availability of detailed questionnaire data regarding breast cancer risk factors, we could control for potential confounding variables. While our physical activity questionnaire was not directly validated, the inverse associations we observed between recent physical activity and BMI and recent physical activity and current cigarette smoking suggest construct validity of our instrument. Furthermore, our physical activity categories classified 49% of women in our study as achieving the current public health recommendation of at least 2.5 hours per week of moderate-to-vigorous physical activity to maintain health [31,32], comparable to the 47.5% of U.S. women estimated to achieve this level of activity in a 2007 national survey [45]. One limitation of our study, however, is that the generalizability of our results may be limited due to the relatively low response proportion to our initial postal questionnaire.

In our cohort of postmenopausal women, we were able to examine the relation of physical activity intensity across the lifespan to postmenopausal breast cancer risk, but we relied upon recall of physical activity during time periods in the distant past. Prior studies have shown reasonably strong correlations between physical activity recalled from 3-5 years [46] and 15 years [47] in the past with physical activity measured objectively at that time, although the validity of physical activity over longer periods of recall is not known. Our observation that physical activity was positively correlated across proximate age periods suggests that our ability to discriminate between certain age periods may have been limited. Furthermore, it is likely that the null associations with historical physical activity in our study resulted from a greater degree of misclassification of historical than recent physical activity.

Additionally, if participants exhibit better recall of vigorous intensity activities than those of lower intensity [48-50], the observation of an association of moderate-to-vigorous physical activity and the lack of an association of light physical activity with breast cancer risk might be explained not by etiological differences, but by misclassification. Yet in our prospectively designed study, exposure measurement error is likely to be non-differential by case status, whereas misclassification of physical activity level in case-control studies has been shown to distort risk estimates as a result of recall bias [51]. It is possible that we did not observe an association of light intensity physical activity at any time period of life with breast cancer risk due to the examples of light activities given in the questionnaire. Our examples of light intensity activities may have reflected a combination of sedentary behaviors (eg: golf [riding in a cart], fishing) and truly light activities (eg: bowling, table tennis, slow walking/slow dancing, light calisthenics, light gardening, horseshoes/croquet, light housework), thereby preventing the assessment of a true association between breast cancer risk and light physical activity [52]. In addition, while providing categories of physical activity duration (<1, 1-3, 4-7, or >7 hours per week) eases the recall burden of participants, such categorization may also permit misclassification if differences in risk exist for more subtle distinctions in the amount of time spent active.

## Conclusion

In summary, our results suggest that moderate-to-vigorous physical activity during later adulthood is associated with reduced postmenopausal breast cancer risk. Future prospective studies designed to assess the relation of physical activity intensity across the lifespan to breast cancer risk will be required to confirm our findings. Although controlled trials or intervention studies are the ideal study designs for disentangling the "dose" of physical activity that may influence breast cancer risk, the cost and duration of such studies for researching the association of physical activity intensity and timing with primary breast cancer limits their feasibility [53]. However, interventions and experimental research will be imperative to investigate the mechanisms by which physical activity intensity and timing influences breast cancer risk.

## List of abbreviations

NIH: National Institutes of Health; AARP: American Association of Retired Persons; ER: estrogen receptor; RR: relative risk; CI: confidence interval; BMI: body mass index; CTS: California Teachers' Study.

## Competing interests

The authors declare that they have no competing interests.

## Authors' contributions

TMP carried out the statistical analysis and drafted the manuscript. TMP, SCM, GLG, NJW, UE, AND MFL participated in the design and coordination of the study. ARH and AS conceived of the study. All authors read and approved the final manuscript.

## Pre-publication history

The pre-publication history for this paper can be accessed here:

http://www.biomedcentral.com/1471-2407/9/349/prepub
